# Supplementary material for: Emr1 regulates the number of foci of the endoplasmic reticulum-mitochondria encounter structure complex
Source: Nat Commun. 2021 Jan 22;12:521. doi: 10.1038/s41467-020-20866-x (PMC7822926; doi:10.1038/s41467-020-20866-x)
Supplement: Supplementary file 1 — Supplementary Information [file 41467_2020_20866_MOESM1_ESM.pdf]

Supplementary Information

Emr1 regulates the number of foci of the endoplasmic reticulum-mitochondria encounter structure complex

Faiz Rasul†, Fan Zheng†, Fenfen Dong†, Jiajia He†, Ling Liu†, Wenyue Liu†, Javairia Yousuf Cheema†， Wenfan Wei†, *, and Chuanhai Fu†, *

† Ministry of Education Key Laboratory for Membrane-less Organelles & Cellular Dynamics, CAS Center for Excellence in Molecular Cell Sciences, Hefei National Laboratory for Physical Sciences at the Microscale, School of Life Sciences, Division of Life Sciences and Medicine, University of Science and Technology of China, 230027 Hefei, P.R. China

* Correspondence should be addressed to Chuanhai Fu or Wenfan Wei: School of Life Sciences, University of Science and Technology of China, Hefei, Anhui, 230027 China; chuanhai@ustc.edu.cn (C.F.); weiwf@ustc.edu.cn (W.W.); Tel. 86 (551) 63600805.

Supplementary information includes five supplementary figures and three tables.

Supplementary Figures

Supplementary Figure 1

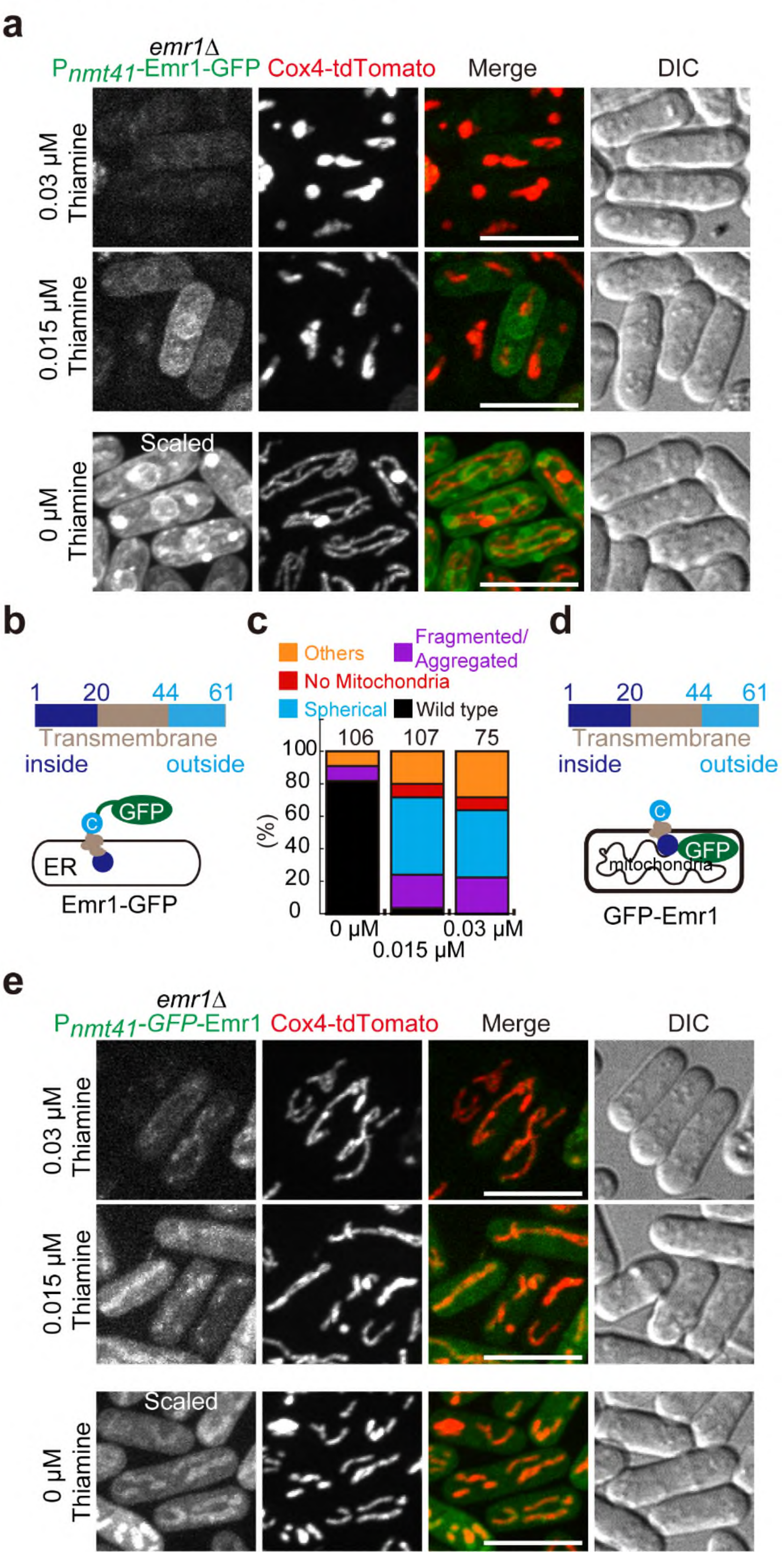

Supplementary Figure 1. Testing the localization of Emr1 that is tagged either at the N-terminus or at the C-terminus.

(a) Maximum projection images of *emr1*Δ cells expressing Cox4-tdTomato and Emr1-GFP (from the *nmt41* promoter). The strain was inoculated into EMM5S media lacking thiamine or containing thiamine at the indicated concentration. The expression levels of Emr1-GFP decreased as the concentration of thiamine increased. Note that tagging Emr1 at its C-terminus enables the fusion protein to localize to the ER and the ER-localizing Emr1-GFP at low expression levels does not rescue the mitochondrial phenotypes caused by the absence of Emr1, indicative of defective function of Emr1-GFP. Scale bar 10 µm.

(b) Diagram illustrating the localization of Emr1-GFP.

(c) Quantification of the indicated mitochondrial phenotypes for the cells in (a). Cell number observed for quantification is shown on the top of the graph.

(d) Diagram illustrating the localization of GFP-Emr1.

(e) Maximum projection images of *emr1*Δ cells expressing Cox4-tdTomato and GFP-Emr1 (from the *nmt41* promoter). The strain was inoculated into EMM5S media lacking thiamine or containing thiamine at the indicated concentration. The expression levels of GFP-Emr1 decreased as the concentration of thiamine increased. Note that tagging Emr1 at its N-terminus enables the fusion protein to localize to mitochondria and the mitochondria-localizing GFP-Emr1 at low expression levels rescues the mitochondrial phenotypes caused by the absence of Emr1, indicating that GFP-Emr1 is functional. Scale bar 10 µm.

Supplementary Figure 2

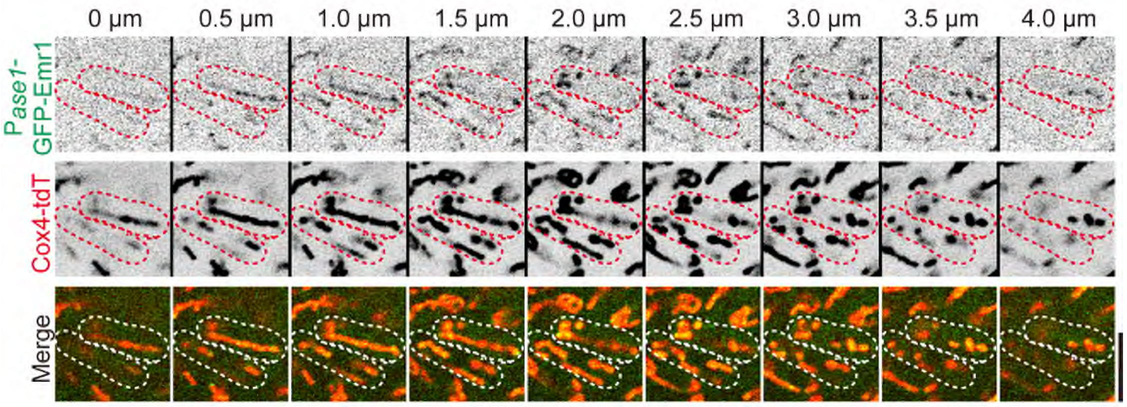

(related to Fig. 2a, bottom panel)

Supplementary Figure 2. Related to main Fig. 2a .

Single-plane images from the Z-stack images of *emr1*Δ cells expressing Cox4-tdTomato and P*ase1*-GFP-Emr1. The axial spacing between each image is 0.5 µm. Dashed lines mark the edge of cells. Scale bar, 10 µm.

Supplementary Figure 3

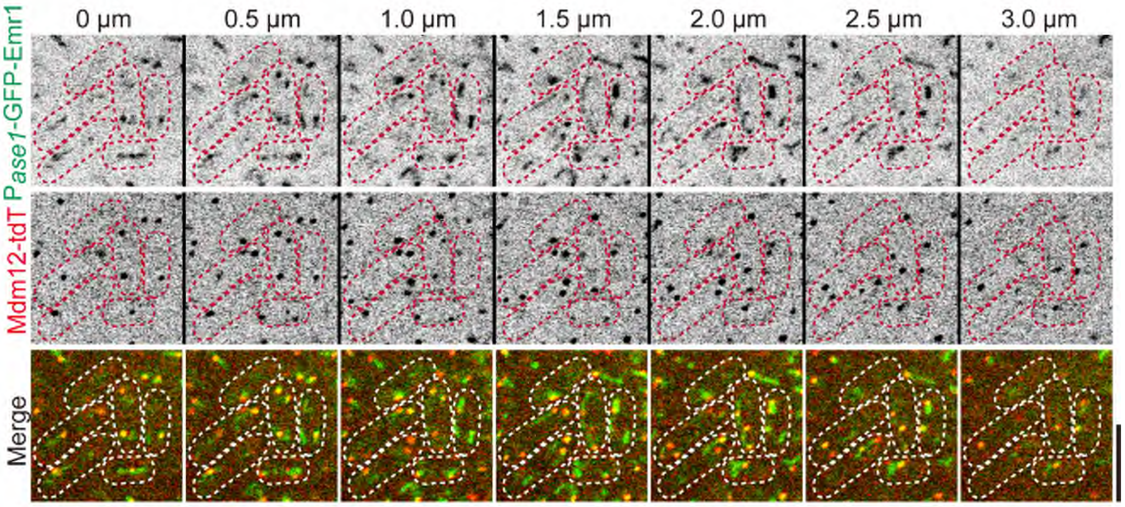

(related to Fig. 4e)

Supplementary Figure 3. Related to main Fig. 4e.

Single-plane images from the Z-stack images of *emr1*Δ cells expressing Mdm12-tdTomato and *Pase1*-GFP-Emr1. The axial spacing between each image is 0.5 µm. Dashed lines mark the edge of cells. Scale bar, 10 µm.

Supplementary Figure 4

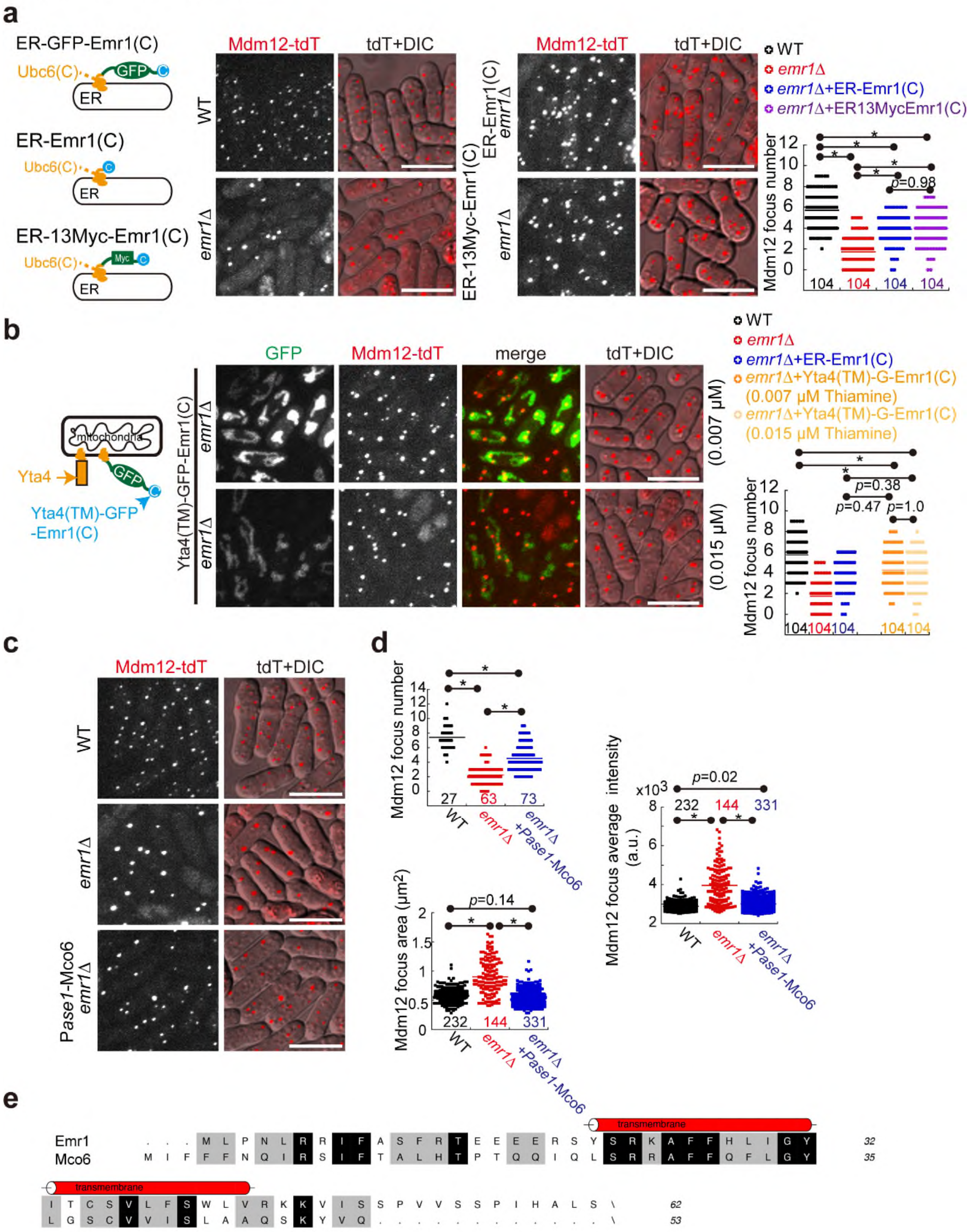

Supplementary Figure 4. Related to main Fig. 6.

(a) Diagrams illustrating the design of ER-GFP-Emr1(C) (also shown in Fig. 6a), ER-Emr1(C), and ER-13Myc-Emr1(C). ER is the ER-localizing fragment from Ubc6 (including its C-terminus (a.a. 226-227) and the adjacent transmembrane domain (a.a. 207-225)). ER-Emr1(C), the Ubc6 fragment is fused to the C-terminus of Emr1 directly; ER-13Myc-Emr1(C), 13Myc is flanked by the Ubc6 fragment and the C-terminus of Emr1. Note that 13Myc is smaller than GFP. Shown on the right are maximum projection images of WT, *emr1*Δ, *emr1*Δ P*nmt41*-ER-Emr1(C), and *emr1*Δ P*nmt41*-ER-13Myc-Emr1(C) expressing Mdm12-tdTomato. Quantification showed that similar to ER-GFP-Emr1(C) (Fig. 6e), ER-Emr1(C) and ER-13Myc-Emr1(C) partially restored the number of Mdm12 foci in *emr1*Δ cells. Statistical analysis was performed by one-way ANOVA ($F(3, 416)=138.28$, $p<0.001$), followed by Tukey honest significance difference test (the *p* values are indicated on the graph; *, $p<0.001$). Cell number observed for quantification is indicated at the bottom of the graph. Scale bar, 10 μm.

(b) Diagram illustrating the design of the chimera Yta4(TM)-GFP-Emr1(C). Yta4(TM) is the N-terminal transmembrane domain of Yta4 (i.e. the first 38 amino acid residues). The chimera (controlled by the *nmt41* promoter) was expressed in *emr1*Δ cells, and cells were cultured in EMM5S media containing either 0.007 μM or 0.015 μM thiamine. Quantification of Mdm12 foci showed that Yta4(TM)-GFP-Emr1(C) partially restores the number of Mdm12 foci in *emr1*Δ cells. For the ease of comparison, the number of mdm12 foci for WT, *emr1*Δ, and *emr1*Δ ER-Emr1(C) cells shown in supplementary Fig. 4a (above), was used. Statistical analysis was performed by one-way ANOVA ($F(4, 518)=100.81$, $p<0.001$), followed by Tukey honest significance difference test (the *p* values are indicated on the graph; *, $p<0.001$). Scale bar, 10 μm.

(c) Expression of Mco6, the budding yeast homolog of Emr1, in *emr1*Δ cells partially restores the number of ERMES foci. Maximum projection images of WT and *emr1*Δ and *emr1*Δ cells expressing Mco6 (from the *ase1* promoter). All cells expressed Mdm12-tdTomato for observation of the ERMES complex. Scale bar, 10 μm.

(d) Quantification of Mdm12-tdTomato focus number, area and average intensity, respectively, in the cells indicated in (c). Statistical analysis was performed by one-way ANOVA (focus number: $F(2, 160)=93.11$, $p<0.001$; focus area: $F(2, 704)=215.95$, $p<0.001$; focus average intensity: $F(2, 704)=210.99$, $p<0.001$), followed by Tukey honest significance difference test (the *p* values are indicated on the graphs; *, $p<0.001$).

(e) Sequence similarity between Emr1 and its budding yeast homolog Mco6. The alignment was performed using ClustalW2. Identical and conserved sequences are colored in black and grey, respectively. The image was created using ALINE [1].

Supplementary Figure 5

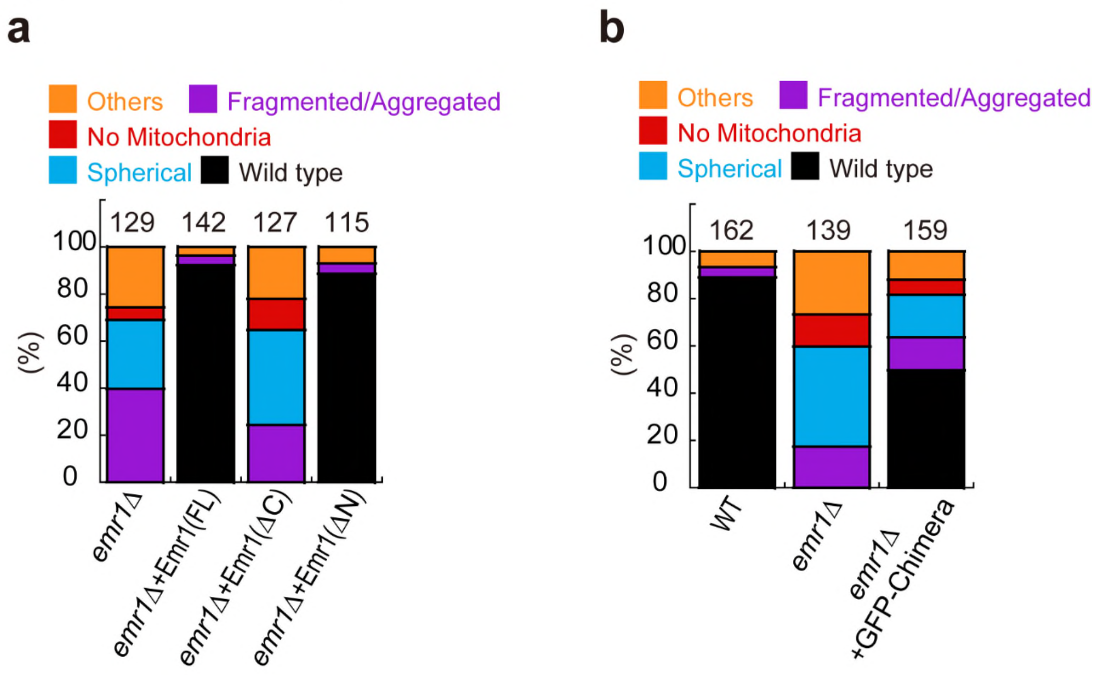

(related to Fig. 6b)

(related to Fig. 7d)

Supplementary Figure 4. Related to main Fig. 6 and Fig. 7.

(a) Quantification of the indicated mitochondrial phenotypes for the cells shown in Fig. 6b. Cell number observed for quantification is shown on the top of the graph.

(b) Quantification of the indicated mitochondrial phenotypes for the cells shown in Fig. 7d. Cell number observed for quantification is shown on the top of the graph.

Supplementary Tables

Table S1. Yeast strains

Strain	Genotype	Source
**Figure 1**		
CF.133	Cox4-GFP:*leu*+ mCherry-Atb2:HygR *ade6*-m210 *ura4*-D18 h-	This study
CF.6844	*emr1*Δ:KanR Cox4-GFP:*leu*+ mCherry-Atb2:HygR *ade6*-m210 *ura4*-D18 h?	This study
CF.6474	WT *ade6*-m210 *leu1*-32 *ura4*-294- h-	This study
CF.6840	*emr1*Δ:KanR *ade6*-m210 *leu1*-32 *ura4*-D18 h+	This study
**Figure 2**		
CF.5003	Cox4-tdTomato:NatR *ade6*-m210 *leu1*-32 *ura4*-D18 h+	This study
CF.8398	*emr1*Δ:KanR Cox4-tdTomato:NatR P*emr1*-GFP-Emr1:*ura*+ *ade6*-m210 *leu1*-32 h?	This study
CF.8159	*emr1*Δ:KanR Cox4-tdTomato:NatR P*ase1*-GFP-Emr1:*leu*+ *ade6*-m210 *ura4*-D18 h?	This study
CF.7697	*emr1*Δ:KanR Cox4-tdTomato:NatR P*nmt41**-GFP-Emr1:*leu*+ *ade6*-m210 *ura4*-D18 h?	This study
CF.9451	*emr1*Δ:KanR *yta4*Δ:KanR P*yta4*-Yta4-13Myc:*ura*+ P*ase1*-GFP-Emr1:*leu*+ *ade6*-m210 *leu1*-32 *ura4*-294 h?	This study
**Figure 3**		
CF.5003	Cox4-tdTomato:NatR *ade6*-m210 *leu1*-32 *ura4*-D18 h+	This study
CF.8414	Cox4-tdTomato:NatR P*nmt41*-GFP-Emr1-ΔN:*leu*+ *ade6*-m210 *ura4*-D18 h+	This study
CF.8418	Cox4-tdTomato:NatR P*nmt41*-GFP-Emr1-ΔC:*leu*+ *ade6*-m210 *ura4*-D18 h+	This study
CF.2378	Cox4-GFP:*leu*+ *ade6*-m210 *ura4*-294 h+	This study
CF.7042	*emr1*Δ:KanR Cox4-GFP:*leu*+ *ade6*-m210 *ura4*-294 h?	This study
CF.8144	*emr1*Δ:KanR Cox4-GFP:*leu*+ P*emr1*-Emr1:*ura*+ *ade6*-m210 *ura4*-294 h?	This study
CF.8145	*emr1*Δ:KanR Cox4-GFP:*leu*+ P*emr1*-Emr1-ΔN:*ura*+ *ade6*-m210 *ura4*-294 h?	This study
CF.8146	*emr1*Δ:KanR Cox4-GFP:*leu*+ P*emr1*-Emr1-ΔC:*ura*+ *ade6*-m210 *ura4*-294 h?	This study
CF.9251	*emr1*Δ:KanR Cox4-GFP:*leu*+ P*ase1*-Mco6:*ura*+ *ade6*-m210 *ura4*-294 h?	This study
CF.6474	WT *ade6*-m210 *leu1*-32 *ura4*-294- h-	This study
CF.6840	*emr1*Δ:KanR *ade6*-m210 *leu1*-32 *ura4*-D18 h+	This study
CF.8150	*emr1*Δ:KanR P*emr1*-Emr1:*ura*+ *ade6*-m210 *leu1*-32 h?	This study
CF.8151	*emr1*Δ:KanR P*emr1*-Emr1-ΔC:*ura*+ *ade6*-m210 *leu1*-32 h?	This study
CF.8152	*emr1*Δ:KanR P*emr1*-Emr1-ΔN:*ura*+ *ade6*-m210 *leu1*-32 h?	This study
**Figure 4**		
CF.4714	Cox4-GFP:*leu*+ Mdm12-tdTomato:NatR *ade6*-m210 *ura4*-D18 h-	This study
CF.8158	*emr1*Δ:KanR Cox4-GFP:*leu*+ Mdm12-tdTomato:NatR *ade6*-m210 *ura4*-D18 h?	This study
CF.8166	*emr1*Δ:KanR Mmm1-tdTomato:NatR P*ase1*-GFP-Emr1:*leu*+ *ade6*-m210 *ura4*-D18 h?	This study
CF.8167	*emr1*Δ:KanR Mdm12-tdTomato:NatR P*ase1*-GFP-Emr1:*leu*+ *ade6*-m210 *ura4*-D18 h?	This study
CF.8168	*emr1*Δ:KanR Sid4-tdTomato:HygR P*ase1*-GFP-Emr1:*leu*+ *ade6*-m210 *ura4*-D18 h?	This study
**Figure 5**		

CF.4712	Cox4-GFP:*leu*+ Mmm1-tdTomato:NatR *ade6*-m210 *ura4*-D18 h-	This study
CF.8157	*emr1*Δ:KanR Cox4-GFP:*leu*+ Mmm1-tdTomato:NatR *ade6*-m210 *ura4*-D18 h?	This study
CF.8166	*emr1*Δ:KanR Mmm1-tdTomato:NatR P*ase1*-GFP-Emr1:*leu*+ *ade6*-m210 *leu1*-32 *ura4*-D18 h?	This study

Figure 6

CF.8158	*emr1*Δ:KanR Cox4-GFP:*leu*+ Mdm12-tdTomato:NatR *ade6*-m210 *ura4*-D18 h?	This study
CF.8399	*emr1*Δ:KanR Cox4-GFP:*leu*+ Mdm12-tdTomato:NatR P*emr1*-Emr1:*ura*+ *ade6*-m210 h?	This study
CF.8400	*emr1*Δ:KanR Cox4-GFP:*leu*+ Mdm12-tdTomato:NatR P*emr1*-Emr1-ΔC:*ura*+ *ade6*-m210 h?	This study
CF.8401	*emr1*Δ:KanR Cox4-GFP:*leu*+ Mdm12-tdTomato:NatR P*emr1*-Emr1-ΔN:*ura*+ *ade6*-m210 h?	This study
CF.5561	Mdm12-tdTomato:NatR *ade6*-m210 *leu1*-32 *ura4*-D18 h+	This study
CF.7024	*emr1*Δ:KanR Mdm12-tdTomato:NatR *ade6*-m210 *leu1*-32 *ura4*-D18 h+	This study
CF.8407	*emr1*Δ:KanR Mdm12-tdTomato:NatR P*nmt41*-GFP-Emr1(C):*leu*+ *ade6*-m210 *ura4*-D18 h+	This study
CF.8423	*emr1*Δ:KanR Mdm12-tdTomato:NatR P*nmt41*-ER-GFP-Emr1(C):*leu*+ *ade6*-m210 *ura4*-D18 h+	This study

Figure 7

CF.5561	Mdm12-tdTomato:NatR *ade6*-m210 *leu1*-32 *ura4*-D18 h+	This study
CF.7024	*emr1*Δ:KanR Mdm12-tdTomato:NatR *ade6*-m210 *leu1*-32 *ura4*-D18 h+	This study
CF.7292	*emr1*Δ:KanR Mdm12-tdTomato:NatR P*nmt41*-GFP-Chimera:*ura*+ *leu1*-32 *ade6*-m210 h?	This study
CF.6477	Cox4-RFP:*leu*+ *ade6*-m210 *ura4*-294 h+	This study
CF.6930	*emr1*Δ:KanR Cox4-RFP:*leu*+ *ade6*-m210 *ura4*-D18 h?	This study
CF.7215	*emr1*Δ:KanR Cox4-RFP:*leu*+ P*nmt41*-GFP-Chimera:*ura*+ *ade6*-m210 h?	This study

Figure 8

CF.6474	WT *ade6*-m210 *leu1*-32 *ura4*-294- h-	This study
CF.6840	*emr1*Δ:KanR *ade6*-m210 *leu1*-32 *ura4*-D18 h+	This study
CF.10032	*psd1*Δ:NatR *ade6*-m210 *leu1*-32 *ura4*-D18 h-	This study
CF.10033	*emr1*Δ:KanR *psd1*Δ:NatR *ade6*-m210 *leu1*-32 *ura4*-D18 h?	This study
CF.10034	*psd2*Δ:NatR *ade6*-m210 *leu1*-32 *ura4*-D18 h+	This study
CF.10038	*emr1*Δ:KanR *psd2*Δ:NatR *ade6*-m210 *leu1*-32 *ura4*-D18 h?	This study
CF.10035	*psd3*Δ:KanR *ade6*-m210 *leu1*-32 *ura4*-D18 h+	This study
CF.10039	*emr1*Δ:KanR *psd3*Δ:KanR *ade6*-m210 *leu1*-32 *ura4*-D18 h?	This study
CF.10036	*ept1*Δ:KanR *ade6*-m210 *leu1*-32 *ura4*-D18 h+	This study
CF.10040	*emr1*Δ:KanR *ept1*Δ:KanR *ade6*-m210 *leu1*-32 *ura4*-D18 h?	This study

Figure S1

CF.7697	*emr1*Δ:KanR Cox4-tdTomato:NatR P*nmt41*-GFP-Emr1:*leu*+ *ade6*-m210 *ura4*-D18 h?	This study
CF.8171	*emr1*Δ:KanR Cox4-tdTomato:NatR P*nmt41*-Emr1-GFP:*leu*+ *ade6*-m210 *ura4*-D18 h?	This study

Figure S2

CF.8159	*emr1*Δ:KanR Cox4-tdTomato:NatR P*ase1*-GFP-Emr1:*leu*+ *ade6*-m210 *leu1*-32 *ura4*-D18 h?	This study
**Figure S3**		
CF.8167	*emr1*Δ:KanR Mdm12-tdTomato:NatR P*ase1*-GFP-Emr1:*leu*+ *ade6*-m210 *leu1*-32 *ura4*-D18 h?	This study
**Figure S4**		
CF.5561	Mdm12-tdTomato:NatR *ade6*-m210 *leu1*-32 *ura4*-D18 h+	This study
CF.7024	*emr1*Δ:KanR Mdm12-tdTomato:NatR *ade6*-m210 *leu1*-32 *ura4*-D18 h+	This study
CF.10029	*emr1*Δ:KanR Mdm12-tdTomato:NatR P*nmt41*-ER-Emr1(C):*leu*+ *ade6*-m210 *leu1*-32 *ura4*-D18 h+	This study
CF.10030	*emr1*Δ:KanR Mdm12-tdTomato:NatR P*nmt41*-ER-13Myc-Emr1(C):*leu*+ *ade6*-m210 *leu1*-32 *ura4*-D18 h+	This study
CF.10028	*emr1*Δ:KanR Mdm12-tdTomato:NatR P*nmt41*-Yta4(TM)-GFP-Emr1(C):*leu*+ *ade6*-m210 *leu1*-32 *ura4*-D18 h+	This study
CF.9252	*emr1*Δ:KanR Mdm12-tdTomato:NatR P*ase1*-Mco6:*ura*+ *ade6*-m210 *leu1*-32 h?	This study
**Figure S5**		
CF.8158	*emr1*Δ:KanR Cox4-GFP:*leu*+ Mdm12-tdTomato:NatR *ade6*-m210 *ura4*-D18 h?	This study
CF.8399	*emr1*Δ:KanR Cox4-GFP:*leu*+ Mdm12-tdTomato:NatR P*emr1*-Emr1:*ura*+ *ade6*-m210 h?	This study
CF.8400	*emr1*Δ:KanR Cox4-GFP:*leu*+ Mdm12-tdTomato:NatR P*emr1*-Emr1-ΔC:*ura*+ *ade6*-m210 h?	This study
CF.8401	*emr1*Δ:KanR Cox4-GFP:*leu*+ Mdm12-tdTomato:NatR P*emr1*-Emr1-ΔN:*ura*+ *ade6*-m210 h?	This study
CF.6477	Cox4-RFP:*leu*+ *ade6*-m210 *ura4*-294 h+	This study
CF.6930	*emr1*Δ:KanR Cox4-RFP:*leu*+ *ade6*-m210 *ura4*-D18 h?	This study
CF.7215	*emr1*Δ:KanR Cox4-RFP:*leu*+ P*nmt41*-GFP-Chimera:*ura*+ *ade6*-m210 h?	This study

Table S2. Plasmids

Plasmid	Genotype	Source
**Figure 2**		
pCF.3292	pJK210-P*emr1*-GFP-Emr1	This study
pCF.3234	pJK148-P*ase1*-GFP-Emr1	This study
pCF.3178	pJK148-P*nmt41*-GFP-Emr1	This study
pCF.3132	pJK210-P*yta4*-Yta4-13Myc	This study
**Figure 3**		
pCF.3230	pJK210-P*emr1*-Emr1	This study
pCF.3231	pJK210-P*emr1*-Emr1-ΔN	This study
pCF.3232	pJK210-P*emr1*-Emr1-ΔC	This study
pCF.3479	pJK210-P*ase1*-Mco6	This study
pCF.3312	pJK148-P*nmt41*-GFP- Emr1-ΔN	This study
pCF.3313	pJK148-P*nmt41*-GFP-Emr1-ΔC	This study

**Figure 4**		
pCF.3234	pJK148-P*ase1*-GFP-Emr1	This study
pPT.111	pGEX-4T-2	This study
pCF.3473	pET22b-Mdm12-GST	This study
pCF.3474	pET22b-Mdm34-GST	This study
pCF.3478	pET28-His-eGFP-Emr1	This study
**Figure 5**		
pCF.3234	pJK148-P*ase1*-GFP-Emr1	This study
**Figure 6**		
pCF.3230	pJK210-P*emr1*-Emr1	This study
pCF.3231	pJK210-P*emr1*-Emr1-ΔN	This study
pCF.3232	pJK210-P*emr1*-Emr1-ΔC	This study
pCF.3285	pJK148-P*nmt41*-GFP-Emr1 (C)	This study
pCF.3302	pJK148-P*nmt41*-ER-GFP-Emr1 (C)	This study
**Figure 7**		
pCF.3068	pJK210-P*nmt41*-GFP-ChiMERA	This study
**Figure S1**		
pCF.3178	pJK148-P*nmt41*-GFP-Emr1	This study
pCF.3026	pJK210-P*nmt41*-Emr1-GFP	This study
**Figure S2**		
pCF.3234	pJK148-P*ase1*-GFP-Emr1	This study
**Figure S3**		
pCF.3234	pJK148-P*ase1*-GFP-Emr1	This study
**Figure S4**		
pCF.3479	pJK210-P*ase1*-Mco6	This study
pCF.3860	pJK148-P*nmt41*-ER-Emr1 (C)	This study
pCF.3861	pJK148-P*nmt41*-ER-13Myc-Emr1 (C)	This study
pCF.3863	pJK148-P*nmt41*-Yta4(TM)-GFP-Emr1 (C)	This study

Table S3. Oligoes

Oligo #	Name	Sequence
oCF.3476	Emr1(N+Tm a.a.1-43) deletion & GSX5 addition in pCF.3178_ F	ggaagtggaagtggaagtggaagtTGGCTGGTGCGAAAAAAGGTGATTTCC
oCF.3477	Emr1 (N+Tm aa.1-43) deletion & GSX5 addition in pCF.3178_ R	acttccacttccacttccacttccGCGGCCGCAAGCTTTGTATAGTTCATC
oCF.3488	Ubc6 Tm+C(619-684) in pCF.3285_F	TGTTTTTTGCCCTTGCTTTAGCTCGTTTTTTTGGAGCTGATTCTagtaaaggagaagaacttttc

oCF.3489	Ubc6 Tm+C(619-684) in pCF.3285/86_R	GCTAAAGCAAGGGCAAAAAACACCAAAACTGCTATGCTCCACATctcgaggccaatttaacaaag
oCF.4251	GFP deletion from pCF.3302_F	tttggagctgattctGCTTGCGGCCGCGGAAGTGGAAGTGGAAGTG
oCF.4252	GFP deletion from pCF.3302_R	tccgcggccgcaagcAGAATCAGCTCCAAAAAAACGAGCTAAAGCA
oCF.4627	Yta4 N+Tm(aa. 1-38) in pjk148-GFP_F	GTAGTGTCTTCTCCTATACATGCGTTATCGTAAtagtcgacccgggtaaaaggaatgtct
oCF.4628	Yta4 N+Tm(aa. 1-38) in pjk148-GFP_R	AGAAGACACTACAGGTGAGGAAATCACCTTTTTtttgtatagttcatccatgcc

References

1. Bond, C.S. & Schuttelkopf, A.W. ALINE: a WYSIWYG protein-sequence alignment editor for publication-quality alignments. *Acta Crystallogr D Biol Crystallogr* **65**, 510-512 (2009).
