# Supplementary material for: Emr1 regulates the number of foci of the endoplasmic reticulum-mitochondria encounter structure complex
Source: Nat Commun. 2021 Jan 22;12:521. doi: 10.1038/s41467-020-20866-x (PMC7822926; doi:10.1038/s41467-020-20866-x)
Supplement: Supplementary file 3 — Reporting Summary [file 41467_2020_20866_MOESM3_ESM.pdf]

nature research

Corresponding author(s): Chuanhai Fu, Wenfan Wei

Last updated by author(s): Nov 27, 2020

Reporting Summary

Nature Research wishes to improve the reproducibility of the work that we publish. This form provides structure for consistency and transparency in reporting. For further information on Nature Research policies, see our Editorial Policies and the Editorial Policy Checklist.

Statistics

For all statistical analyses, confirm that the following items are present in the figure legend, table legend, main text, or Methods section.

n/a	Confirmed	
☐	☒	The exact sample size (*n*) for each experimental group/condition, given as a discrete number and unit of measurement
☐	☒	A statement on whether measurements were taken from distinct samples or whether the same sample was measured repeatedly
☐	☒	The statistical test(s) used AND whether they are one- or two-sided<br>*Only common tests should be described solely by name; describe more complex techniques in the Methods section.*
☒	☐	A description of all covariates tested
☒	☐	A description of any assumptions or corrections, such as tests of normality and adjustment for multiple comparisons
☐	☒	A full description of the statistical parameters including central tendency (e.g. means) or other basic estimates (e.g. regression coefficient) AND variation (e.g. standard deviation) or associated estimates of uncertainty (e.g. confidence intervals)
☐	☒	For null hypothesis testing, the test statistic (e.g. *F*, *t*, *r*) with confidence intervals, effect sizes, degrees of freedom and *P* value noted<br>*Give P values as exact values whenever suitable.*
☒	☐	For Bayesian analysis, information on the choice of priors and Markov chain Monte Carlo settings
☒	☐	For hierarchical and complex designs, identification of the appropriate level for tests and full reporting of outcomes
☒	☐	Estimates of effect sizes (e.g. Cohen's *d*, Pearson's *r*), indicating how they were calculated

Our web collection on statistics for biologists contains articles on many of the points above.

Software and code

Policy information about availability of computer code

Data collection	For imaging data collection, Volocity (version 6.1.1.) was used. For collection of western blot data, ChemiScope (Model No. 3300 Mini from CLiNX Science Instruments) was used.
Data analysis	Imaging data were analyzed with MetaMorph (version 7.7) and ImageJ (version 1.52i), graphs and plots were generated with KaleidaGraph (version 4.5), statistical analysis was performed with KaleidaGraph (version 4.5). ClusterW2 was used for sequence alignment. ALINE (version 1.0.025) was used to create the image of sequence alignment. TMHMM 2.0 was used to predict the transmembrane domain of Emr1.

For manuscripts utilizing custom algorithms or software that are central to the research but not yet described in published literature, software must be made available to editors and reviewers. We strongly encourage code deposition in a community repository (e.g. GitHub). See the Nature Research guidelines for submitting code & software for further information.

Data

Policy information about availability of data

All manuscripts must include a data availability statement. This statement should provide the following information, where applicable:

- Accession codes, unique identifiers, or web links for publicly available datasets
- A list of figures that have associated raw data
- A description of any restrictions on data availability

All data generated or analyzed during this study are included in this published article (and its supplementary information files). All protein and gene sequences of Schizosacchromyces pombe were obtained from the database Pombase (https://www.pombase.org). The sequence of Mco6 was obtained from the Saccharomyces Genome Database (SGD) (https://www.yeastgenome.org). The strains and plasmids used in this study are readily available upon request. All other information that support the findings of this paper are available upon reasonable request.

Field-specific reporting

Please select the one below that is the best fit for your research. If you are not sure, read the appropriate sections before making your selection.

☒ Life sciences ☐ Behavioural & social sciences ☐ Ecological, evolutionary & environmental sciences

For a reference copy of the document with all sections, see nature.com/documents/nr-reporting-summary-flat.pdf

Life sciences study design

All studies must disclose on these points even when the disclosure is negative.

Sample size	No sample-size calculation was performed. In general, strains to be tested were cultured and imaged in parallel and >100 cells/foci (except Fig. 7e and supplementary Fig. 4d (Mdm12 foci)) were selected randomly from the acquired images.<br><br>The absence of Emr1 or Emr1 mutants affect the number, size, and fluorescent intensity of Mdm12 and Mmm1 so significantly that the effects were seen directly from the microscopic images (i.e. Figs. 4a, 5a, 6b, 6d, and 7b) before quantification. Therefore, we simply performed quantification with cells, as many as possible, obtained from parallel experiments. Generally, a large number of cells were able to be collected from one microscopic experiment due to the small size of yeasts. Consistent with the microscopic observations, statistical analysis gave rise to very small p values between the groups showing difference in the microscopic images (i.e. Figs. 4c, 5c, 6c, 6e, 7c, and 7e). Given the consistence between the statistical data and direct microscopic observations, the sample sizes are sufficient.
Data exclusions	No data were excluded from the analyses.
Replication	Imaging data presented in this study were repeated two times, and the results were similar. For data obtained by western blotting analysis, the experiments were also performed at least two times, and the results were similar. These statements have been added in the method section under Statistics and Reproducibility.
Randomization	Each maximum projection image contains many yeast cells and yeast cells were selected randomly for the analyses.
Blinding	During collection of microscopy data, strain number/names were generally used as file names for saving data. Therefore, the investigators were not blinded to group allocation during data collection. Similarly, when opening files for data analysis, the investigators knew the origin of the data. Therefore, the investigators were not blinded to group allocation during data analysis. This did not affect the outcomes of data analysis because experiments were performed in parallel and cells from microscopic images were chosen randomly for analysis.

Reporting for specific materials, systems and methods

We require information from authors about some types of materials, experimental systems and methods used in many studies. Here, indicate whether each material, system or method listed is relevant to your study. If you are not sure if a list item applies to your research, read the appropriate section before selecting a response.

Materials & experimental systems

n/a	Involved in the study	
☐	☒	Antibodies
☒	☐	Eukaryotic cell lines
☒	☐	Palaeontology and archaeology
☒	☐	Animals and other organisms
☒	☐	Human research participants
☒	☐	Clinical data
☒	☐	Dual use research of concern

Methods

n/a	Involved in the study	
☒	☐	ChIP-seq
☒	☐	Flow cytometry
☒	☐	MRI-based neuroimaging

Antibodies

Antibodies used	Anti-tdTomato (Mouse, Origene, #TA180009), Anti-GFP (Goat, Rockland, #600-101-215), Anti-GFP (Rabbit; raised by Genscript, www.genscript.com.cn), Anti-Myc (Mouse, Invitrogen, #13-2500), Anti-tubulin (Rabbit, BioAcdemia, #63-160), Anti-His (Mouse, Abclonal, #WH080027/AE003), Anti-GST (Rabbit, Abclonal, #WH080027/AE006), Anti-Mti2 (Rabbit) is a gift from the laboratory of Prof. Huang Ying (indicated in the article https://doi.org/10.1111/febs.15021), Goat Anti-Mouse (GAM)-HRP Conjugate (Bio-RAD, #170-5047), Goat anti-Rabbit HRP Conjugate (Bio-RAD, #170-5046), HRP Rabbit Anti-Goat (Abclonal, #AS029).
Validation	Most of the antibodies used in this study are antibodies against tags (i.e. GFP, tdTomato, Myc, GST, and His). These tag antibodies specifically detected tag-fused proteins at the positions on gels where tag-fused proteins with the predicted size were expected, confirming the reliability of the tag antibodies. The anti-Mti2 antibody has been used in the published work https://doi.org/10.1111/febs.15021. The anti-tubulin antibody has been validated by BioAcademia with the whole cell extracts of S.pombe (see https://www.bioacademia.co.jp/en/product_img/409/E63-160%20anti-betatublin%20_S.%20pombe_antibody,%20WB,%20IF.pdf).
